# Electronic device and social network use and sleep outcomes among adolescents: the EHDLA study

**DOI:** 10.1186/s12889-023-15579-x

**Published:** 2023-05-19

**Authors:** Anelise Reis Gaya, Rodolfo Brum, Keith Brites, Adroaldo Gaya, Letícia de Borba Schneiders, Miguel Angelo Duarte Junior, José Francisco López-Gil

**Affiliations:** 1 Federal University of Rio Grande do Sul (UFRGS), Rua Felizardo, n° 750 - Jardim Botânico, Porto Alegre, RS Brazil; 2 Universidad Autónoma de Madrid, Madrid, Spain; 3 Universidad Pública de Navarra, IdiSNA, Navarrabiomed, Hospital Público de Navarra, Pamplona, Spain; 4 Department of Environmental Health, T.H. Chan School of Public Health, Harvard University, Boston, MA USA; 5 One Health Research Group, Universidad de Las Américas, Quito, Ecuador

**Keywords:** Sleep quality, Physical activity, Online Social Networks, Lifestyle, Youths, Teenagers

## Abstract

**Background:**

Considering the evident risk in the literature between the use of screen devices and sleep, there are still few studies on the relationship between each electronic screen device, media programs and sleep duration and sleep-related problems among adolescents and which variables interfere in these relationships. Therefore, this study has the following objectives: (1) to determine which are the most common electronic display devices related to sleep time and outcomes and (2) to determine which are the most common social network applications, such as Instagram and WhatsApp, associated with sleep outcomes.

**Methods:**

This was a cross-sectional study with 1101 Spanish adolescents between 12 and 17 years old. Age, sex, sleep, psychosocial health, adherence to the Mediterranean diet (MD), sport practice, and time spent on screen devices were assessed by an ad hoc questionnaire. Linear regression analyses were applied, adjusting for several covariables. Poisson regression was applied between the sexes. A *p* value < 0.05 was considered statistically significant.

**Results:**

Cell phone use was more associated with sleep time (13%). In boys, time spent on cell phones (prevalence ratio [PR] = 1.09; *p* < 0.001) and videogames (PR = 1.08; *p* = 0.005) had a higher prevalence ratio. When psychosocial health was included in the models, we found the greatest association (Model 2: PR = 1.15; *p* = 0.007). For girls, time spent on the cell phone was significantly associated with sleep-related problems (PR = 1.12; *p* < 0.001), and adherence to the MD became the second most important in the model (PR = 1.35; *p* < 0.001), followed by psychosocial health and cell phone use (PR = 1.24; *p* = 0.007). Time spent on WhatsApp was associated with sleep-related problems only among girls (PR = 1.31; *p* = 0.001) and was the most important variable in the model along with MD (PR = 1.26; *p* = 0.005) and psychosocial health (PR = 1.41; *p* < 0.001).

**Conclusions:**

Our results suggest a relationship between cell phones, video games, and social networks with sleep-related problems and time.

## Introduction

Children and adolescents have shown insufficient sleep time and several sleep disorders [1, 2]. Sleep outcomes are one of the most important concerns associated with the health of children and adolescents worldwide [3, 4]. Duration, quality of sleep and analyses of several disorders while sleeping are reported as risk factors for physical and mental concerns [5–7]. Scientific evidence has provided results about the early consequences of unhealthy sleep outcomes in children and adolescents’ development [1–4, 8].

Concerning several biological, social, and environmental factors associated with sleep outcomes [4], daily time spent on physical activity, sports practice, and sedentary behaviors have been identified as modifiable risk factors for improving sleep time and sleep-related problems in German and American adolescents [9–11]. The advances in technology inserted in the intense routine of work and studies, as well as leisure, contribute to the increase in the time spent on screen devices and, consequently, to the shorter time involved in physical activities, according to a systematic review carried out with data from children and adolescents from 12 countries [7]. However, the role of all sedentary time and specific television (TV) time on unhealthy sleep outcomes has already been documented with emphasis on studies about the optimal time of sleep for a healthy profile [2, 9, 10, 12].

Children and adolescents spent approximately eight hours per day in sedentary behaviors after and before the COVID-19 pandemic, and the amount of time recommended by Canadian guidelines for screen time for the young population is less than two hours a day [10, 13–15]. Studies have appointed the role of total sedentary time, sitting time, TV time, and videogames as the main behaviors linked with obesity, physical activity, sleep, headaches, and other health complications, such as mental, social, and cognitive disturbances [7, 9, 13]. However, there are gaps in the literature regarding the relatively new electronic devices among adolescents, such as cell phones, tablets, and computers. In addition, the relationships of these electronic devices used outside of school or related during homework with sleep duration and sleep-related problems are still little explored [12, 13]. This is probably the main specific sedentary behavior associated with sleep time and sleep-related problems such as difficulty sleeping and waking up, interruption, time in bed, and sleep-related problems [12, 16].

Evidence points to more factors related to sleep; young people who manage to maintain an adequate diet and psychosocial health status, free of stress and emotional stress, may have sleep outcomes favorable to health in relation to their peers. It is expected that children who engage in physical activities and/or sports on a daily basis are protected from the risk associated with a sedentary profile in sleep outcomes [3, 9, 17]. This is because physical activity is a protective behavior and is inversely associated with the early development of health risk factors in the young population, such as obesity and insulin resistance [7]. Thus, despite several studies including sleep outcomes and physical activity, we still do not know if and how regular sports practices could be associated with more desirable sleep outcomes [3, 9]. Therefore, it appears to be interesting to understand which devices - excluded time used for study or homework - and what children do when they are on these screen electronic devices. In addition, we aimed to understand whether there is a relationship between each of these screen electronic devices and specific social network apps with sleep duration and sleep-related problems, considering sports practice, body mass index, age, sex, socioeconomic status, and psychosocial health in this possible association. The high exposure of adolescents in situations of social and mental stress, in contact with a blue light screen and exposure to radiofrequency electromagnetic fields is common in electronic screen source devices, and this exposure is associated with sleep disorders [12, 18]. Recently, Cabré-Riera et al. [12] found that evening time in front of electronic devices and exposure to radiofrequency electromagnetic field mobile phones are associated with objective sleep measurements and disorders. However, they did not find any association between the use of devices during the day and sleep outcomes. There are few studies on the relationship between each electronic screen device, media programs and sleep duration and sleep-related problems among adolescents.

Thus, with the aim of contributing to healthier sleep outcomes in youth, our study has three main purposes: [1] to determine the most common electronic screen devices related to sleep outcomes and [2] to determine which are the most common social network apps, such as Instagram and WhatsApp, associated with sleep outcomes. We hypothesized that adolescents spend almost all their time on cell phones, are usually involved with social network apps, spend less time sleeping and have more sleep-related problems.

## Materials and methods

### Study design and participants

Adolescents (n = 1101) from three secondary schools (CE *El Ope*, IES *Vicente Medina*, and IES *Pedro Guillén*) in *Valle de Ricote* (Region of Murcia, Spain) aged 12–17 years of both sexes were evaluated in this cross-sectional study. All assessments regarding sociodemographic, lifestyle, and health-related factors were obtained in the school environment, specifically during physical education classes, at the beginning of the 2021/2022 school year. All participants, as well as their parents or guardians, received a term indicating their interest and authorization to participate in this study.

Adolescents who were exempt from the physical education discipline were excluded from the study, considering that the evaluations would be carried out during the discipline’s class time, adolescents who had some contraindication with medical evidence that prevented them from performing physical activities were also excluded (e.g., physical injuries, cardiovascular diseases), students who were undergoing pharmacological treatment, and students who did not present the informed consent form. This study obtained ethical approval from the Bioethics Committee of the University of Murcia (ID 2218/2018) and the Ethics Committee of the Albacete University Hospital Complex and the Albacete Integrated Care Management (ID 2021-85). This study was carried out in accordance with the Declaration of Helsinki, respecting the human rights of all participants involved. The present study is part of a large project entitled “The Eating Healthy and Activities of Daily Living (EHDLA) Study”, which evaluated 1378 adolescents as a representative sample. All secondary schools from the Valle de Ricote (Region of Murcia, Spain) (i.e., three) were assessed for this study. This study involves adolescents using a simple random sampling technique. For the present study, only adolescents who had complete data on the analyzed variables were included: electronic device and social network use, sleep duration and sleep-related problems, organized sports participation, adherence to the Mediterranean diet (MD), psychosocial health, and anthropometric variables. The detailed methodology of the EHDLA study has been published elsewhere [19].

### Procedures

#### Electronic device and social network use (independent variables)

Electronic device and social network use was assessed through a self-report questionnaire, and adolescents declared the time they spent on different screen-based sedentary activities. The questions answered correspond to the days of the week and the days of the weekend: “How many hours a day, in your free time, do you usually spend watching TV, playing video games (not including movement or fitness games), using your computer and cell phone?”. In addition, the following question was used: “How many hours a day in your free time do you normally spend using social networks? This question was asked individually for Instagram and WhatsApp. A weighted summation of responses was performed (i.e., five days a week and two days a weekend) and presented as latent mean hours [19].

#### Sleep duration and sleep-related problems (dependent variables)

Sleep duration was assessed by asking adolescents about weekdays and weekend days separately, with the following questions: “What time do you usually go to bed?” and “What time do you usually wake up?”. The mean daily sleep duration was calculated as follows: [(mean nighttime sleep duration on weekdays x 5) + (average nighttime sleep duration on weekends x 2)]/7. Sleep-related problems were assessed using the BEARS scale (B = Bedtime Issues, E = Excessive Daytime Sleepiness, A = Night Awakenings, R = Regularity and Duration of Sleep, S = Snoring), a tool used to track sleep disorders most common in the population young, from a clinical interview [20]. Questions related to sleep, such as problems at bedtime, excessive daytime sleepiness, waking during the night, regularity and duration of sleep, and snoring, were evaluated. This instrument was validated in a Spanish version to screen for sleep-related problems in the pediatric population [21]. Adolescents were categorized as “at least one sleep-related problem” and “no sleep-related problems”.

### Covariates

#### Sociodemographic variables

Age and sex were self-reported by adolescents. Socioeconomic status was assessed with the Family Affluence Scale (FAS-III) [22].

#### Anthropometric variables

The adolescents’ body weight was measured using an electronic scale (with a precision of 0.1 kg) (Tanita BC-545, Tokyo, Japan), and height was determined using a portable height rod with a precision of 0.1 cm (Leicester Tanita HR 001, Tokyo, Japan). Body mass index (BMI) was calculated as the ratio of body weight (in kg) to height (in meters squared).

#### Organized sports participation

Information on organized sports participation was carried out through specific questions such as “Do you attend an organized sports club?” (yes or no).

#### Psychosocial health

The one-sided self-assessed version of the Strengths and Difficulties Questionnaire (SDQ) was applied to assess different behavioral, emotional, and social problems related to psychosocial health in the young population [23]. For this study, the Spanish version of the SDQ [24, 25] was used. The questionnaire contains 25 items divided into 5 different subscales: emotional problems, conduct problems, hyperactivity, problems with colleagues, and prosocial behavior. A 3-point Likert scale was used with different options: not true (0), somewhat true [1] or certainly true [2]. The score for each subscale ranges from 0 to 10 points. The first four subscales (Emotional Problems, Conduct Problems, Hyperactivity, and Peer Problems) determine an overall score for psychosocial problems. The fifth subscale (prosocial behavior) assesses resources rather than problems and is conceptually different from the assessment of psychosocial problems. Therefore, their score was not included in the total score of psychosocial problems [23].

#### Adherence to the mediterranean diet

The Mediterranean Diet Quality Index for Children and Teenagers (KIDMED) was used to evaluate adherence to the Mediterranean diet (MD). This is a validated instrument that is widely used in the young Spanish population [26, 27]. The KIDMED index ranges from − 4 to 12 and is based on a 16-question test, in which it seems that reported healthy characteristics of the MD are scored + 1 and unhealthy items are scored − 1. The sum of all scores was categorized into three different levels: high MD (> 8 points); moderate MD (4 to 7 points); and low MD (< 3 points) [26]. For further analyses, we collapsed into optimal MD (high MD) or nonoptimal MD (moderate MD and low MD).

### Statistical analysis

For sample characterization, data were expressed as the mean and standard deviation for continuous variables and frequency and percentage for categorical variables. Linear regression analyses were conducted to test the association between electronic device or social network use and sleep duration. Data were expressed as unstandardized beta values (*B*) with 95% confidence intervals (95% CI) and standardized beta values (*β*). Conversely, Poisson regression analyses were conducted to test the association between electronic device or social network use and sleep-related problems. Data are expressed as the prevalence ratio (PR) and 95% CI. Sex, age, body mass index, organized sport participation, psychosocial health, and adherence to the MD were considered covariates. The association between independent variables or covariates and dependent variables was assessed by Spearman’s rho (*ρ*). Variables that did not show a statistically significant correlation (i.e., *p* > 0.05) were not included in the models. Since sex and sleep-related problems were correlated (*p* < 0.05), the results of the Poisson regression were stratified by sex. The best model was chosen considering both the AIC (Akaike information criterion) and the BIC (Bayesian information criterion). A *p* value < 0.05 was considered significant for all analyses. All analyses were performed using the Statistical Package for the Social Sciences (SPSS) (IBM Corp, Armonk, NY, USA) version 27.0 for Windows.

## Results

Most adolescents did not participate in organized sports participation (66.3%) and did not report optimal adherence to the MD (62.4%). The average socioeconomic status was predominant in this sample (53.0%). Regarding sleep-related problems, 58.9% of adolescents reported at least one sleep-related problem. The most commonly used electronic device is the cell phone (mean = 3.6 ± 1.1 h per day). Adolescents reported a high time spent using both Instagram (mean = 3.5 ± 1.1 h per day) and WhatsApp (mean = 3.5 ± 1.3 h per day) (Table 1).

**Table 1:** Descriptive data of the study participants

	N (%)	Mean	SD
Total	1101 (100.0)		
Age (years)		14.11	1.55
BMI (kg/m^2^)		22.77	4.82
Weight (kg)		60.06	15.25
Height (cm)		161.88	8.77
Sex (%)			
Boys	504 (45.8)		
Girls	597 (54.2)		
Socioeconomic status (%)			
Low	236 (21.4)		
Medium	583 (53.0)		
High	282 (25.6)		
Organized sport participation (%)			
No	729 (66.3)		
Yes	371 (33.7)		
SDQ (%)			
Normal	524 (73.1)		
Borderline	97 (13.5)		
Abnormal	96 (13.4)		
Adherence to the MD (%)			
Optimal MD	355 (37.6)		
Nonoptimal MD	589 (62.4)		
Sleep duration (minutes)		488.43	56.03
Sleep-related problems (%)	1101 (100.0)		
No sleep-related problems	452 (41.1)		
At least one sleep-related problem	649 (58.9)		
Computer use (hour)		2.2	1.2
Videogames use (hour)		2.2	1.3
TV use (hour)		2.3	1.0
Cell phone use (hour)		3.6	1.1
Instagram (hour)		3.5	1.1
WhatsApp (hour)		3.5	1.3

BMI: body mass index; MD: Mediterranean diet; SDQ: Strengths and Difficulties Questionnaire; TV: television; SD: standard deviation.

Table 2 shows the crude and composite linear regression model including time spent on cell phones, computers, Instagram, WhatsApp, and all variables that were previously associated with sleep duration (Spearman’s rho: *p* < 0.05). Regarding the main objective of our study, the electronic device that showed the highest association with sleep time was the cell phone (*β* = -0.306; *p* < 0.001). This model explained approximately 20% of the variation in sleep time. In relation to social networks, Instagram and WhatsApp social media programs both adjusted models explained approximately 13% of the variation in sleep time. In both models, Instagram (*β* = -0.145; *p* < 0.001) showed a slightly higher association with sleep time in comparison with WhatsApp (*β* = -0.131; *p* = 0.001).

**Table 2:** Association of all electronic devices and social network use with sleep time

Sleep duration				
	*R*²	*B* (95% CI)	*β*	*p*
**Model 1**	0.11			
Cellphone use (time)		-16.49 (-21.47, -14.70)	-0.336	< 0.001
**Model 2**	0.20			
Cellphone use (time)		-14.56 (-17.94, -11.22)	-0.306	< 0.001
Age (years)		-7.68 (-10.17, -5.20)	-0.211	< 0.001
Organized sports participation (Ref. No)				
Yes		9.12 (1.01, 17.23)	0.076	0.028
SDQ (Ref. Normal)				
Borderline		-15.68 (-22.89, -0.40)	-0.010	0.006
Abnormal		-21.28 (-31.37, -8.98)	-0.129	< 0.001
**Model 3**	0.01			
Computer use (time)		-5.07 (-7.79, -2.34)	-0.110	< 0.001
**Model 4**	0.12			
Computer use (time)		-4.80 (-7.95, -1.64)	-0.107	0.003
Age (years)		-8.95 (-11.52, -6.38)	-0.248	< 0.001
Organized sports participation (Ref. No)				
Yes		10.86 (2.41, 19.30)	0.092	0.012
SDQ (Ref. Normal)				
Borderline		-18.74 (-30.33, -7.15)	-0.115	0.002
Abnormal		-23.87 (-35.54, -12.21)	-0.146	< 0.001
Diet (Ref. Optimal MD)				
Nonoptimal MD		-8.54 (-16.60, -0.45)	-0.075	0.039
**Model 5**	0.05			
Instagram use (time)		-9.70 (-12,77, -6.63)	-0.230	< 0.001
**Model 6**	0.13			
Instagram use (time)		-6.03 (-9.15, -2.96)	-0.145	< 0.001
Age (years)		-7.85 (-10.51, -5.19)	-0.220	< 0.001
Organized sports participation (Ref. No)				
Yes		10.80 (2.34, 19.23)	0.092	0.012
SDQ (Ref. Normal)				
Borderline		-14.70 (-26.42: -2.98)	-0.090	0.014
Abnormal		-20.46 (-32.18: -8.75)	-0.126	0.001
Diet (Ref. Optimal MD)				
Nonoptimal MD		-9.11 (-17.16, -1.06)	-0.081	0.027
**Model 7**	0.02			
WhatsApp use (time)		-6.65 (-10.38, -2.91)	-0.131	0.001
**Model 8**	0.13			
WhatsApp use (time)		-5.60 (-9.19, -2.01)	-0.112	0.002
Age (years)		-8.76 (-11.36, -6.17)	-0.245	< 0.001
Organized sports participation (Ref. No)				
Yes		10.80 (2.32, 19.27)	0.092	0.013
SDQ (Ref. Normal)				
Borderline		-17.30 (-18.23, -5.58)	-0.106	0.004
Abnormal		-23.76 (-35.43, -12.10)	-0.146	< 0.001
Diet (Ref. Optimal MD)				
Nonoptimal MD		-10.26 (-18.31, -2.21)	-0.091	0.013

MD: Mediterranean diet; SDQ: Strengths and Difficulties Questionnaire. Data are expressed as nonstandardized beta values with their 95% confidence intervals, standardized beta values, and *p* values.

Table 3 shows the raw and composite Poisson regression model including all electronic devices and social networks and several other independent variables previously associated with sleep-related problems (Spearman’s rho: *p* < 0.05). The best model was chosen considering both the AIC and the BIC. As we experienced a significant interaction between sex and electronic devices in these models, data were presented separately for boys and girls. For boys, the time spent on the cell phone was associated with problems related to sleep, both educationally (PR = 1.09; *p* = 0.001) and within the model (PR = 1.15; *p* = 0.007), videogames (PR = 1.08; *p* = 0.005) and the computer (PR = 1.06; *p* = 0.03) showed associations in an educational way. However, when the SDQ was included in all models, we found the highest association with sleep-related problems (*p* < 0.001). For girls, the time spent playing videogames (PR = 1.07; *p* < 0.001) and using the cell phone showed a significant association with problems related to sleep in an educational way (PR = 1.12; *p* < 0.001) and with the other variables of the model (PR = 1.07; *p* = 0.02). Time spent on WhatsApp was associated with sleep-related problems only among girls in a basic way (PR = 1.31; *p* < 0.001) and within the model (PR = 1.20; *p* = 0.02).

**Table 3:** Association of all electronic devices and social network programs with sleep-related problems

	Sleep-related problems					
	Boys			Girls		
	**AIC**	PR (95%CI)	*p*	**AIC**	PR (95%CI)	*p*
**Model 1**	**852.2**			**1088.0**		
Cellphone use (time)		1.09 (1.02, 1.18)	0.001		1.12 (1.06, 1.19)	< 0.001
**Model 2**	**485.2**			**746.8**		
Cellphone use (time)		1.15 (1.04, 1.28)	0.007		1.07 (1.00, 1.14)	0.03
Age (years)		0.91(0.84, 0.99)	0.04		1.01 (0.97, 1.05)	0.55
Organized sports participation (Ref. No)						
Yes		1.13 (0.88, 1.46)	0.30		1.09 (0.93, 1.29)	0.25
SDQ (Ref. Normal)						
Borderline		1.69 (1.26, 2.26)	< 0.001		1.35 (1.17, 1.55)	< 0.001
Anormal		1.57 (1.21, 2.04)	< 0.001		1.35 (1.16, 1.56)	< 0.001
Diet (Ref. Optimal MD)						
Nonoptimal MD		1.12 (0.87, 1.45)	0.34		1.24 (1.06, 1.46)	0.007
**Model 3**	**1093.5**			**1093.5**		
Computer use (time)		1.06 (1.00, 1.13)	0.03		1.03 (0.98, 1.08)	0.20
**Model 4**	**496.0**			**747.4**		
Computer use (time)		1.01 (0.92, 1.10)	0.78		1.03 (0.98, 1.09)	0.17
Age (years)		0.93 (0.85, 1.01)	0.10		1.02 (0.97, 1.06)	0.33
Sports participation (Ref. No)						
Yes		1.16 (0.90, 1.48)	0.24		1.11 (0.94, 1.31)	0.20
SDQ (Ref. Normal)						
Borderline		1.83 (1.37, 2.45	< 0.001		1.38 (1.20, 1.59)	< 0.001
Abnormal		1.59 (1.21, 2.10)	< 0.001		1.37 (1.19, 1.59)	< 0.001
Diet (Ref. Optimal MD)						
Nonoptimal MD		1.18 (0.91, 1.53)	0.19		1.27 (1.08, 1.49)	0.003
**Model 5**	**851.7**			**1091.1**		
Videogames use (time)		1.08 (1.02, 1.15)	0.005		1.07 (1.03, 1.12)	< 0.001
**Model 6**	**487.5**			**747.7**		
Videogames use (time)		1.07 (0.99, 1.16)	0.08		1.02 (0.96, 1.08)	0.43
Age (years)		0.93 (0.86, 1.01)	0.11		1.02 (0.98, 1.06)	0.29
Organized sports participation (Ref. No)						
Yes		1.15 (0.90, 1.48)	0.25		1.10 (0.93, 1.30)	0.23
SDQ (Ref. Normal)						
Borderline		1.82 (1.38, 2.41)	< 0.001		1.37 (1.18, 1.58)	< 0.001
Abnormal		1.67 (1.26, 2.22)	< 0.001		1.38 (1.19, 1.59)	< 0.001
Diet (Ref. Optimal MD)						
Nonoptimal MD		1.16 (0.91, 1.49)	0.22		1.27 (1.08, 1.49)	0.003
**Model 7**	**519.1**			**740.3**		
WhatsApp use (time)		1.12 (0.76, 1.76)	0.56		1.31 (1.16, 1.48)	0.001
**Model 8**	**480.2**			**728.5**		
WhatsApp use (time)		1.12 (0.74, 1.69)	0.56		1.20 (1.02, 1.42)	0.02
Age (years)		0.92 (0.85, 1.01)	0.10		1.01 (0.97, 1.05)	0.54
Organized sports participation (Ref. No)						
Yes		1.13 (0.88, 1.35)	0.33		1.12 (0.94, 1.33)	0.18
SDQ (Ref. Normal)						
Borderline		1.80 (1.33, 2.45)	< 0.001		1.34 (1.16, 1.54)	< 0.001
Abnormal		1.65 (1.12, 2.17)	< 0.001		1.41 (1.22, 1.16)	< 0.001
Diet (Ref. Optimal MD)						
Nonoptimal MD		1.20 (0.93, 1.54)	0.14		1.26 (1.07, 1.48)	0.005

AIC: Arkaike information criterion, BIC: Bayesian information criterion, MD: Mediterranean diet, PR: prevalence ratio SDQ: Strengths and Difficulties Questionnaire. Data are expressed as prevalence ratios values with their 95% confidence intervals, and *p* values.

## Discussion

The aim of this study was to verify the relationship of different types of electronic devices, different types of social network use excluding time use for study or homework and organized sports participation with adequate time and sleep-related problems in a sample of Spanish adolescents. The main results support the notion that high time spent in cell phones and, specifically, in social network programs showed an association with a reduction in time and sleep-related problems. Despite these main results, organized sports participation showed an association with longer sleep duration. However, we did not find an interaction with electronic devices and social networks.

In our study, adolescents spent approximately ten hours per day in electronic devices, including TV, computers, cell phones, and videogames, and of this time spent approximately seven hours in social network programs over the day. These results carry an interesting finding because when we adjusted our models for psychosocial health, diet, age, sex, cell phone, computers, and videogames showed an association with sleep outcomes. Daily time spent on TV did not show associations. In fact, in several countries around the world, cell phones are becoming the electronic device most used by children and adolescents instead of TV time [28].

Despite the most common relationship of different electronic devices with sleep time [11, 12, 28] the results also suggest that cell phones are associated with sleep-related problems, followed by playing videogames in boys and longer time spent on WhatsApp in girls. In our study, sleep-related problem variables included questions about bedtime problems, regularity and duration of sleep, excessive daytime sleepiness, sleep-disordered breathing, awakening during night, and nightmares. Thus, our findings suggest that adolescents’ sleep-related problems seem to also be conditioned by daily time spent in cell phones, videogames, and social networks in girls [10, 11] and showed several differences between boys and girls. For example, playing videogames did not show an association with sleep-related problems in girls. However, time spent in social networks seems to be a predictor of sleep-related problems among girls. In the present study, 58.9% of adolescents showed insufficient sleep-related problems, results that agree with previous studies [28] and become deeply important to understand this relationship.

In fact, the results corroborate other studies and confirm that more than half of adolescents do not sleep well and may have different consequences of poor sleep outcomes. Regardless of the discrepancy in numbers caused by the different methods used to measure sleep outcomes, studies point to the same scenario and show a greater number of adolescents who do not sleep well and do not have sleep-related problems [10–12].

Furthermore, because only cell phones, videogames, and WhatsApp were electronic devices associated with adolescents’ sleep-related problems, we speculate that these associations could be explained by the specific features, qualities, and characteristics of each electronic device. From this, it is important to highlight that we found differences between electronic devices in relation to sex when associated with sleep-related problems. Additionally, it seems important to highlight that the predictors associated with sleep time were not the same as those associated with sleep-related problems. This suggests the necessity of a better understanding of the mechanisms between these variables [29].

In fact, several mechanisms have been consistently appointed as an explanation of the association between social networks and sleep outcomes. Some impact has been linked with a higher exposure to blue light that could be associated with circadian rhythm modification. Furthermore, the other mechanisms appointed must be related to the quality of activities in each social network program [29, 30]. In this way, Cabré-Riera et al. [12] recently showed that preteenagers with high evening whole-brain radiofrequency electromagnetic field doses from cell phones had shorter total sleep times. In the same sense, Miligi [31] reported several studies in adults that showed encephalogram alterations induced by radiofrequency electromagnetic field mobile phones. This study appointed a hypothesis associated with several brain conditions associated with time of use and perhaps with the features of cell phone activities. However, most studies are necessary to understand which kind of social network users have sleep outcomes affected and which kind of sleep conditions are affected.

The results also drew attention because participation in organized sports did not show an association with sleep time and did not show interaction with any electronic device. Several physiological responses of physical activity on sleep outcomes have been shown [9]. However, the results seem to be inconsistent [3, 9, 11]. As in the present study, several studies appointed some evidence that a longer sleep duration was associated with less sedentary time and a higher proportion of daily physical activity. In this way, the importance of being active in relation to sleep-related problems is highlighted [3, 9, 11, 17].

On the other hand, our data appointed psychosocial health and age as the main predictors among adolescents associated with sleep outcomes, while diet was only associated with sleep-related problems in girls. Thus, our data indicate the necessity of a deeper understanding of the bidirectional relationship between mental health and sleep disorders since childhood. This is worrying, considering that adolescents with psychopathologies have presented sleep disorders. Several studies have shown that even in adolescents without psychiatric disorders, sleep-related problems have been associated with episodes of depression, anxiety, and change of mood in youth [4].

Adolescents and young adults are the largest users of social media and other technologies. Social media platforms have benefits such as the engagement and dissemination of healthy practices but also harm such as longer sedentary behavior, longer sleep latency, and affected mental health [32]. In a review carried out by Alonzo et al. [33], five cohort studies demonstrated that excessive use of social networks is a risk factor for poor sleep-related problems during follow-up. In addition, among these studies, risk associations were highlighted between excessive computer use and sleep disturbances in boys and cell phone use with inverse associations in relation to sleep duration during the week and sleep compensation due to greater cell phone time on weekends [34].

The review by Alonzo et al. [33] identified that studies with representative samples from Europe and Asia and studies with convenience samples from China and the USA present risk associations between social networks and higher sleep-related problems [35]. In Switzerland, nocturnal use of social media was associated with shorter sleep duration and with sleep-related problems, suggesting that the use of social media and mobile devices are indicators that stand out for future interventions in adolescents when the subject is the pattern and sleep quality.

The relationship between the use of new technologies and mental health has demonstrated that there are other linking factors for this relationship, one of which is the practice of physical activity. The fact that the use of social networks can negatively influence health is simply because the long time spent using devices could be dedicated to the practice of physical activities or organized sports participation [36]. Therefore, the implications focused on perceptions of reducing risks to mental health and general health in adolescents should prioritize interventions that aim to replace time in sedentary activities, such as the use of devices and social networks, with time in physical activities [37].

In addition, diet was associated with sleep-related problems in girls and proved to be an important variable in the models when relating mobile screen devices to sleep time. Perhaps because we have bidirectional conditions, diet seems to be an important predictor associated with sleep disorders [38] and could show an important role between excess weight and sleep-related problems [39]. These results are in line with evidence found by previous studies [38, 40] that highlighted different associations between variables of eating habits and sleep outcomes. Similarly, one previous systematic review concluded that longer sleep durations appear to be associated with healthier dietary patterns [39]. However, this systematic review pointed out that this association seems to be affected by several confounding factors, such as sex and excess weight [39].

In fact, it is necessary to consider the sleep profile as a predictor for a healthy lifestyle. Health professionals must pay attention to this behavior and start to include it as an approach at school, for example [2, 41]. Other than that, the results appointed for the necessity of intervention studies including reduction of leisure time spent in electronic devices [1]. Sleep outcomes, including both time and quality, have been appointed as the most important behavior associated with global health [4]. Thus, in addition to spending a great amount of time in sedentary behaviors and not participating in organized sports participation, adolescents in fact do not show adherence to the sleep guidelines and already do not have sleep-related problems, suggesting an unhealthy profile in this age phase [42]. The results suggest that there may be a modification of adolescents in their choice and time on an electronic device. TV may be becoming the least used device and giving way to the cell phone as the main focus of intervention in relation to the greater amount of time spent during the day [43]. Independent of all other important factors, psychosocial health, diet, and cell phone use seem to be the main predictors of sleep duration.

However, the present study has several limitations. Although our sample has many evaluated adolescents, we present cross-sectional results, and we were not able to understand the causal relationship between sleep outcomes and electronics devices, which could of course be bidirectional. Likewise, sleep-related problems and time spent in electronic devices and social networks were measured by a questionnaire, which could be underestimated by adolescents. Finally, the use of questionnaires may also be a limitation of our study, which presumes the risk of participant recall bias. However, the strength of our study is that to our knowledge, this is the first study that appointed the impact of cell phones and not television on sleep time and sleep-related problems and the great impact of social network programs on sleep-related problems in adolescents.

## Conclusion

Our results suggest a relationship between cell phones, playing video games and the use of social networks in time in free time with problems related to sleep and sleep time. The association of social networks with sleep outcomes pointed to the importance of better understanding what kind of activities teenagers are engaged in that may be linked to sleep-related problems. In addition, it is important to emphasize the need for young people to become more active because, on average, young people spend their entire day away from school on their cell phones. These findings contribute to the literature in terms of boosting the development of new studies focused on time spent on social networks and not just in front of screens. The type of content accessed and the relationships developed through social networks can have a strong impact not only on sleep-related problems but also on the general health of adolescents.

## Data Availability

Sets used and/or analyzed during the current study are available from the corresponding author based on reasonable query data.

## Abbreviations

AIC: Akaike information criterion, BIC:Bayesian information criterion
BIC: Bayesian information criterion
MD: Mediterranean diet
PR: prevalence ratio
SDQ: Strengths and Difficulties Questionnaire
TV: Television.
